# Modulation at Age of Onset in Tunisian Huntington Disease Patients: Implication of New Modifier Genes

**DOI:** 10.1155/2014/210418

**Published:** 2014-09-01

**Authors:** Dorra Hmida-Ben Brahim, Marwa Chourabi, Sana Ben Amor, Imed Harrabi, Saoussen Trabelsi, Marwa Haddaji-Mastouri, Moez Gribaa, Sihem Sassi, Fatma Ezzahra Gahbiche, Turkia Lamouchi, Soumaya Mougou-Zereli, Sofiane Ben Ammou, Ali Saad

**Affiliations:** ^1^Department of Cytogenetics and Reproductive Biology, Farhat HACHED Hospital, Ibn El Jazzar Road, 4000 Sousse, Tunisia; ^2^Department of Neurology, Sahloul Hospital, Sahloul Road, 4054 Sousse, Tunisia; ^3^Department of Epidemiology and Medical Statistics, Farhat HACHED Hospital, Ibn El Jazzar Road, 4000 Sousse, Tunisia

## Abstract

Huntington's disease (HD) is an autosomal dominant neurodegenerative disorder. The causative mutation is an expansion of more than 36 CAG repeats in the first exon of *IT15* gene. Many studies have shown that the *IT15* interacts with several modifier genes to regulate the age at onset (AO) of HD. Our study aims to investigate the implication of CAG expansion and 9 modifiers in the age at onset variance of 15 HD Tunisian patients and to establish the correlation between these modifiers genes and the AO of this disease. Despite the small number of studied patients, this report consists of the first North African study in Huntington disease patients. Our results approve a specific effect of modifiers genes in each population.

## 1. Background

Huntington disease (HD) is an autosomal dominant disorder caused by the expansion of polymorphic CAG repeat in the coding sequence of* IT15* (OMIM no. 143100) also called huntingtin (*htt*) gene that leads to a progressive loss of neurons preferentially in the striatum and cortex. The symptoms, which usually appear between 40 and 50 years of age, are cognitive defects, psychiatric disorders, and motor dysfunction [1]. More than 36 repeats can cause the disease with the age at onset (AO) being inversely related to CAG repeat number on expanded chromosome. However, variation in CAG repeat number alone explains only around 60% of the variability in AO [2]. Evidence has been provided for genetic modifiers as well as for environmental factors that affect the AO. Several well-powered studies clearly implicate environmental modifiers in the AO and progression of HD [3]. Many genomic variations have also been tested for their influence on AO. Possible candidates are genes encoding products interacting with wild type or mutant* huntingtin*. Polymorphisms in these genes, exerting no effects in unaffected individuals, could modify the course of disease. Several studies have shown an effect of the unexpanded CAG repeat of the* IT15* gene [4]. Other studies suggested a contribution of the gene variations of the N-methyl-D-aspartate receptor (NMDAR) subtypes NR2A (*GRIN2A*) (OMIM no. 6139971) and NR2B (*GRIN2B*) (OMIM no. 613970) to critically influence the variability in AO [2]. Many other modifier genes have also been tested for their influence on AO among them the polymorphic (Gln-Ala) repeat in the transcriptional coactivator CA150 (or* TCERG1* gene) (OMIM no. 605409) [5] and the CAG repeat expansion at the TATA-Box binding protein (*TBP*) (OMIM no. 607136), which is a factor of transcription [6].

In the present study we analyzed 4 polymorphisms that have not yet been described in modulation of AO in HD: CAG repeat of the* DRPLA* gene (OMIM no. 125370), CTG repeat of the* DMPK* (OMIM no. 160900), CAG repeat of the* ATXN1* (OMIM no. 164400) gene, and CTG repeat of the* JPH3* (OMIM no. 606438) implicated in Huntington's disease-like-2 (HDL2), a phenocopy of Huntington's disease. We also investigated 6 polymorphisms that are already known to be associated with HD: rs1969060 in* GRIN2A*, rs890 in* GRIN2B*, CAG repeat at* TBP* gene, (Gln-Ala) repeat in* TCERG1*, and unexpanded and expanded CAG repeat of the* IT15* gene.

## 2. Material and Methods

### 2.1. HD Patients

Our study population consisted of 15 Tunisian unrelated patients with clinical diagnosis of HD. All patients gave their consent for all information to be published. This research paper consists of a retrospective study that does not require ethics committee approval at our institution.

For all patients, the age-at-onset was estimated as the age when motor or cognitive symptoms were first noticed. The age-at-onset ranged from 24 to 72 years with a mean age-at-onset of 44 years. Informed consent was obtained from all patients. HD CAG repeat sizes were determined by polymerase chain reaction using an assay counting the perfectly repeated (CAG) units. Repeated numbers of patients derived from European institutions were randomly checked in our laboratory with a reference control. The expanded CAG repeats ranged from 40 to 49 trinucleotide repeats. The median repeat number was 44.

### 2.2. DNA Samples

Genomic DNA was isolated from 5 mL of peripheral blood with the flexiGen DNA kit from Qiagen according to manufactured protocol and then diluted to a final concentration of 200 ng/*μ*L.

### 2.3. Genotyping of Polymorphic Repeats

Polymorphic repeats in* htt*,* DMPK*,* DRPLA*,* ATXN1*,* JPH3*,* TBP*, and* TCERG1* genes were determined using a modification of the PCR amplification assay reported by Warner et al., with fluorescent oligonucleotide primers flanking the repeats detailed in Table 1 [7]. Determination of the number of repeats was carried out by fragment analysis with the ABI 310 Genetic Analyzer System (Applied Biosystems) according to the manufacturer's instructions. PCR conditions are available upon request.

### 2.4. Genotyping of* GRIN2A* and* GRIN2B*

Genotyping for SNP rs1969060 (*GRIN2B*) and SNP rs890 (*GRIN2A*) was performed by direct sequencing (see Table 1). PCR conditions are available upon request.

### 2.5. Statistical Analysis

Statistical analysis was performed using SPSS 17.0. A possible modifying effect on the HD age-at-onset of the respective polymorphisms was investigated by applying a model of analysis regression with multiple variables. The goodness of fit was evaluated by the proportion of variation in the age-at-onset, explained by the coefficient of determination (*R* ^2^). For analysis, variance in the age-at-onset for the CAG repeats in* htt* was determined alone as well as in addition to different polymorphisms. A change of *R* ^2^ indicated a relative improvement of the model of multiple regressions when the respective factors were added to the effect of the expanded* huntingtin* allele (Δ*R* ^2^). This identified the percentage of the variance that was attributable to the candidate modifier genes, when there was a significant *P* value. A *P* value of less than 0.05 was considered significant.

Correlation between respective polymorphic repeats lengths and the AO was performed using the Pearson correlation coefficient (*r*). As adopted for the coefficient of determination (*R* ^2^), a *P* value of less than 0.05 was considered as significant.

## 3. Results

Both negative correlation and significant effect of the expanded CAG repeat number in the* huntingtin* gene on the AO of HD patients have been shown in numerous studies [3, 8]. In our study, we could also confirm these observations by applying a statistical multiple regression model of an analysis of variance (Table 2). CTG repeat in the* JPH3* gene was also found to be inversely correlated to the AO (see Table 2).

To investigate the possible modifiers that can influence the AO, we followed a multiple regression model. The value of *R* ^2^ was determined for genotypes at each individual locus together with the CAG repeat number on expanded chromosomes. This value was then compared with the value of *R* ^2^ obtained by considering expanded CAG repeat alone. A change of *R* ^2^ (Δ*R* ^2^) is the measure of the influence of putative modifying factors on the variation of AO (Table 3).

Using this model in HD, the value of *R* ^2^ was 0.745 (*P* = 0.000). Thus, for HD 74.5% of the variation of AO can be explained by expanded CAG repeats.

We also established the implication of other polymorphisms studied in* DRPLA*,* DMPK*,* TCERG1*,* TBP, ATXN1*,* JPH3*,* GRIN2B* (rs890), and* GRIN2A* (rs1969060) genes in the unexplained variance of AO (see Table 3).

## 4. Discussion

The evaluation of the age-at-onset in HD presents a challenge that has to be solved as precise as possible. In this pilot study, we characterized a large number of polymorphisms in genes that are suggested to act as possible modifiers for the AO of HD. Environmental modifiers, already known to have a considerable effect on AO and progression of HD, were not investigated in the present study. It would be of great interest to also look for environmental modifiers in African population, where environmental factors may differ between countries and thus be highly informative. Furthermore, via gene-environment interactions, environmental modifiers may affect the detectability of genetic modifiers in specific cohorts. Wexler [3] reported that 60% of the variance remaining in AO is attributable to environmental modifiers in Venezuelan population.

Genetic modifier factors have been indicated in HD as the length of the disease causing expanded polyglutamine tract in* huntingtin* explains only 65–70% of the variance in the age-at-onset [2]. In our report, the CAG repeat accounts for 74.5% of the variance in AO which is in accord with other previous studies [9]. Previous studies have established that the unexpanded HD allele has also been shown to have some association with OA in 754 patients from North America, Europe, and Australia [10]. Later AO was being associated with longer repeats lengths in normal HD gene [10], whereas a study of 138 patients from Wales found negative correlation between the normal CAG repeat and the AO (*P* = 0.014) [11]. Other studies did not confirm any correlation [2, 8, 12]. We have found a negative correlation between unexpanded CAG and AO but, that is, not significant. This result can be explained by the low number of patients. The combination with the expanded allele, using the model of analysis regression, indicated elevation of value of *R* ^2^ from 0.745 to 0.789 (*P* = 0.000); this elevation indicated that 4.4% of the total variability in the AO was attributable to the variations in CAG normal repeats in the huntingtin gene. Thus, we could interpret 17.18% of the unexplained variation in AO by variation at this locus.

Several data have suggested that the NMDA subset of glutamate receptor can contribute to neurodegeneration in HD. These receptors permit influx of calcium and, when activated, can generate neuronal death. NMDARs are multimeric complexes composed of NR1 subunits together with NRA2, NR2B, NR2C, and/or NR2D. In the cortex and striatum, NR2A and NR2B predominate; moreover the NR2B/NR2A ratio is higher in the striatum than other brain regions [13]. Both NR2A and NR2B subunits are encoded, respectively, by* GRIN2A* and* GRIN2B* genes. A study with 167 patients from Germany interested to investigate the association between the AO and two SNPs: a C/T SNP (rs1969060) in* GRIN2A* and a T/G SNP (rs890) in* GRIN2B*, showed that 4.5% of the unexplained variation in AO could be interpreted by a C/T SNP and 2.31% of the unexplained variation in OA could be contributed to the T/G SNP [13]. A Venezuelan study was conducted on the same number of samples and was interested in these two polymorphisms and indicates that 4.5% of the unexplained variation in AO could be attributed to the* GRIN2A* gene, although the* GRIN2B* gene had no implication in the variation of the AO [2].

Here, we found that the polymorphism studied in the* GRIN2A* added to the CAG expansion shows a low variation from 0.745 to 0.748; we might explain that 1.17% of the unexplained variation in AO can be associated to this polymorphism. When the* GRIN2B* polymorphism added the regression model, the value of *R* ^2^ increases (from 0.7.45 to 0.765). This data can explain that 7.81% of the unexplained variation in AO was attributable to the T/G SNP in the* GRIN2B* gene.

Some previous studies have indicated an association of the* TBP* gene with HD. This gene is a good candidate as the encoded protein forms insoluble aggregates in the nucleus of neuronal cells in HD patients [4]. Similar to huntingtin, TBP contains a polymorphic CAG repeat; mutant huntingtin interacts with TBP and impairs the functional conformation of the transcription factor [14]. In this report, the *R* ^2^ statistic increases slightly from 0.745 to 0.747 with the TBP genotypes included. This low variation might explain that 0.78% of the unexplained variation in AO can be contributed to this locus.

Age at onset in HD might be influenced by the length of the polymorphic (Gln-Ala) repeat in* TCERG1* gene. This gene encodes the transcriptional coactivator CA150, the human homologue of a Caenorhabditis elegans protein that interacts with n-terminal fragments of huntingtin [2]. The study of 432 American patients and another of 427 Venezuelan patients demonstrated that this locus can influence the AO, respectively, at 1.07% and 2.19% [2, 15]. In this present report, when we added the* TCERG1* genotypes in the model of regression analysis, the *R* ^2^ statistic rose from 0.744 to 0.746. This increase indicates that 0.78% of the unexplained variance of the AO in HD could be explained by variation of imperfect (Gln-Ala) repeat at* TCERG1* locus. This strong variability of results between different populations may suggest a specific effect of modifiers genes in each population.

We are also interested in investigating the implication of four novel genes: CAG polymorphic repeat of the* DRPLA* and* ATXN1* genes and CTG polymorphic repeat of the* JPH3* and* DMPK* genes.* DRPLA*,* ATXN1*,* DMPK*, and* JPH3* are responsible, respectively, for the dentatorubral-pallidoluysian atrophy disorder (DRPLA), spinocerebellar ataxia type 1 (SCA1), myotonic dystrophy 1 (DM1), and Huntington's disease-like-2 (HDL2). As it is the case with HD, DRPLA, SCA1, and DM1 are all caused by a gain of function mechanism [16, 17]. Similar to HD, the HDL2 major symptom is chorea. Based on those arguments we conducted the analysis of the 4 novel polymorphisms to look for their involvement in the variation in AO.

Here, we found that* JPH3* CTG repeat was inversely correlated to the AO (see Table 2); this correlation was significant at *P* = 0.043. Given that HD is uncommon in our country,* JPH3* finding needs to be improved by studies in larger international cohorts.

Using the model of regression analysis, we could confirm the implication of* DRPLA*,* DMPK*,* ATXN1*, and* JPH3* in the variability of AO in HD. For the* DRPLA* gene, we noticed an increase in the *R* ^2^ value from 0.745 to 0.793. Thus, we indicated that 18.75% of the unexplained variation in AO might be influenced by the variation in this locus. When we added the* DMPK* gene to the HD expanded allele we observed that *R* ^2^ rose slightly from 0.745 to 0.750. This increase allows interpreting 1.95% of the unexplained variation in AO by this gene. In our sample, we can interpret 14.45% of the unexplained variation in AO by the variation of the CAG repeat in the* ATXN1* gene (*R* ^2^ rose from 0.745 to 0.782). Finally, we have established the involvement of the polymorphic CTG repeat in the* JPH3* gene at 11.32% of the unexplained variation in AO (*R* ^2^ rose modestly from 0.745 to 0.774).

## 5. Conclusion

In conclusion, the objective of all studies is to obtain a complete understanding of the variance in OA, that is, attributable to genetic factors other than the length of the unexpanded repeat in the* IT15* gene. We report here the first North African study on Huntington disease. Despite the poor knowledge of HD epidemiological distribution in North African population, we succeeded to prove the implication of 9 polymorphisms encompassing 4 novel ones located at* DRPLA*,* DMPK*,* ATXN1*, and* JPH3* genes. Our results suggest a specific Tunisian age of onset prediction model that should be tested in larger Tunisian HD population.

## Figures and Tables

**Table 1:** Studied polymorphisms and corresponding oligonucleotide primers.

Genes	Polymorphisms	Primers	
*GRIN2A *	SNP rs890	Forward	5′ TGT ACC CAC ATA TAT ACA GAC AC 3′
		Reverse	5′ TGA GAT GGT AGA TTC CGA CTG A 3′

*GRIN2B *	SNP rs1969060	Forward	5′ GGA GGG TAG AGC GGA GAA AG 3′
		Reverse	5′ TCC TTT CAC AAG CAG TGT GC 3′

*IT15 *	CAG	Forward	5′ ATG AAG GCC TTC GAG TCC CTC AAG TCC TTC 3′
		Reverse	5′TCA GCA TCC CAG TTT GAG ACG TGC TGC TGC TGC TGC 3′

*TCERG1 *	Gln-Ala	Forward	5′ CAG AAC TGA CAC CTA TGC TTG C 3′
		Reverse	5′TCA GCA TCC CAG TTT GAG ACG CTT GCA CTT GTG CCT GGA C 3′

*TBP *	CAG	Forward	5′ GAC CCC ACA GCC TAT TCA GA 3′
		Reverse	5′TCA GCA TCC CAG TTT GAG ACG TGC TGC TGC TGC TGC 3′

*JPH3 *	CTG	Forward	5′CCC AGG AAT CTC GTC TTT CA 3′
		Reverse	5′TCA GCA TCC CAG TTT GAG ACG AGC AGC AGC AGC AGC 3′

*ATXN1 *	CAG	Forward	5′ TGG AGG CCT ATT CCA CTC TG 3′
		Reverse	5′TCA GCA TCC CAG TTT GAG ACG TGC TGC TGC TGC TGC 3′

*DMPK *	CTG	Forward	5′ CTC GAA GGG TCC TTG TAG CC 3′
		Reverse	5′TCA GCA TCC CAG TTT GAG ACG AGC AGC AGC AGC AGC 3′

*DRPLA *	CAG	Forward	5′ CAC CCA CCAGTC TCA ACA CA 3′
		Reverse	5′TCA GCA TCC CAG TTT GAG ACG TGC TGC TGC TGC TGC 3′

Fluorescent primer∗			5′ FAM-TCA GCA TCC CAG TTT GAG ACG 3′

*The fluorescent primer is identical to 5′ half (underlined) of reverse primers of all triplet repeat polymorphisms. It was added to the PCR mixture in combination with each pair of triplet repeat nucleotide primers.

**Table 2:** Pearson correlation coefficient between studied polymorphisms and age at onset of HD in our population.

Gene (polymorphism)	Pearson correlation coefficient *r*	*P* value
*IT15* (expanded CAG)	−0.863	0.000∗
*IT15* (unexpanded CAG)	−0.383	0.176
*DRPLA *(CAG)	−0.225	0.440
*DMPK* (CTG)	0.295	0.306
*ATXN1* (CAG)	−0.290	0.315
*TBP* (CAG)	−0.299	0.299
*JPH3* (CTG)	−0.547	0.043∗
*TCERG1* (Gln-Ala)	−0.151	0.606
*GRIN2A* (rs1969060)	0.287	0.320
*GRIN2B* (rs890)	−0.129	0.661

*P < 0.5.

**Table 3:** Linear regression analysis of polymorphisms in candidate genes affecting the age at onset of Huntington disease in Tunisian patients (in addition to the contribution of the expanded CAG repeat (HD CAG)).

Gene (polymorphism)	*R* ^2^	Δ*R* ^2^	% unexplained variability	*P* value
*HD* CAG	0.745	—	—	0.000∗
*HD* CAG + normal CAG	0.789	0.044	17.18	0.000∗
*HD* CAG + *DRPLA* (CAG)	0.793	0.048	18.75	0.000∗
*HD* CAG + *DMPK* (CTG)	0.750	0.005	1.95	0.000∗
*HD* CAG + *ATXN1* (CAG)	0.782	0.037	14.45	0.000∗
*HD* CAG + *TBP* (CAG)	0.747	0.002	0.78	0.001∗
*HD* CAG + *JPH3* (CTG)	0.774	0.029	11.32	0.000∗
*HD* CAG + *TCERG1* (Gln-Ala)	0.746	0.001	0.39	0.001∗
*HD* CAG + *GRIN2A* (rs1969060)	0.748	0.003	1.17	0.000∗
*HD* CAG + *GRIN2B* (rs890)	0.765	0.020	7.81	0.000∗

*P < 0.05.

Variance in AO for the CAG repeats is indicated as well as in combination with the different examined polymorphisms. *R* ^2^ illustrates the relative improvement of the regression model when the various genotypes are considered in addition to the HD CAG repeats. Δ*R* ^2^ values quantify these differences. *P* value refers to *R* ^2^.
